# Field-Emission Scanning Electron Microscope as a Tool for Large-Area and Large-Volume Ultrastructural Studies

**DOI:** 10.3390/ani11123390

**Published:** 2021-11-27

**Authors:** Bogdan Lewczuk, Natalia Szyryńska

**Affiliations:** Department of Histology and Embryology, Faculty of Veterinary Medicine, University of Warmia and Mazury in Olsztyn, Oczapowskiego 13, 10-719 Olsztyn, Poland; natalia.skiepko@uwm.edu.pl

**Keywords:** scanning electron microscope, array tomography, serial block-face imaging, ultrastructure

## Abstract

**Simple Summary:**

Ultrastructural studies of cells and tissues are usually performed using transmission electron microscopy (TEM), which enables imaging at the highest possible resolution. The weak point of TEM is the limited ability to analyze the ultrastructure of large areas and volumes of biological samples. This limitation can be overcome by using modern field-emission scanning electron microscopy (FE-SEM) with high-sensitivity detection, which enables the creation of TEM-like images from the flat surfaces of resin-embedded biological specimens. Several FE-SEM-based techniques for two- and three-dimensional ultrastructural studies of cells, tissues, organs, and organisms have been developed in the 21st century. These techniques have created a new era in structural biology and have changed the role of the scanning electron microscope (SEM) in biological and medical laboratories. Since the premiere of the first commercially available SEM in 1965, these instruments were used almost exclusively to obtain topographical information over a large range of magnifications. Currently, FE-SEM offers many attractive possibilities in the studies of cell and tissue ultrastructure, and they are presented in this review.

**Abstract:**

The development of field-emission scanning electron microscopes for high-resolution imaging at very low acceleration voltages and equipped with highly sensitive detectors of backscattered electrons (BSE) has enabled transmission electron microscopy (TEM)-like imaging of the cut surfaces of tissue blocks, which are impermeable to the electron beam, or tissue sections mounted on the solid substrates. This has resulted in the development of methods that simplify and accelerate ultrastructural studies of large areas and volumes of biological samples. This article provides an overview of these methods, including their advantages and disadvantages. The imaging of large sample areas can be performed using two methods based on the detection of transmitted electrons or BSE. Effective imaging using BSE requires special fixation and en bloc contrasting of samples. BSE imaging has resulted in the development of volume imaging techniques, including array tomography (AT) and serial block-face imaging (SBF-SEM). In AT, serial ultrathin sections are collected manually on a solid substrate such as a glass and silicon wafer or automatically on a tape using a special ultramicrotome. The imaging of serial sections is used to obtain three-dimensional (3D) information. SBF-SEM is based on removing the top layer of a resin-embedded sample using an ultramicrotome inside the SEM specimen chamber and then imaging the exposed surface with a BSE detector. The steps of cutting and imaging the resin block are repeated hundreds or thousands of times to obtain a z-stack for 3D analyses.

## 1. Introduction

The first transmission electron microscope (TEM) was invented by Max Knoll and Ernst Ruska at the Technische Hochschule zu Berlin in 1931 [1] based on research on electron motion in a magnetic field and the possibility of focusing the electron beam by Hans Busch [2]. This instrument created the opportunity to overcome the barrier of resolution occurring in light microscopy; however, the method of studying the internal organization of animal cells required much time and the development of specimen preparation methods and commercially available electron microscopes, which could be used in biological laboratories. In the 1940s, 1950s, and 1960s, scientists developed protocols for animal tissue fixation and preparation for TEM studies, which enabled images of satisfactory quality to be obtained [3,4,5,6]. As a gold standard, aldehydes were introduced for the fixation of proteins and osmium tetroxide for the preservation of lipids and contrasting of membranes [3,4,5]. The development of ultramicrotomy allowed the preparation of ultrathin sections that were essential for obtaining high-quality images in terms of resolution and focus [7,8]. The contrasting of sections using lead citrate and uranyl acetate improved the differentiation of the structures [9,10,11]. Moreover, significant progress occurred in the construction of the TEM, as electron guns, electromagnetic lenses, and power supplies became more stable and efficient. The simplification of TEM operation for use by unskilled operators was also important. From the end of the 1960s, transmission electron microscopy could provide high-quality, high-resolution images of animal specimens. The 70s and 80s of the last century was a period of intensive research on animal cells’ and tissues’ ultrastructure. Further development of transmission electron microscopy included introduction of cryo-techniques, immunochemistry, and digital imaging [12,13,14,15,16].

The main weakness of transmission electron microscopy is the limited ability to analyze the ultrastructure of large areas and volumes of biological samples. Although the introduction of motorized stages and digital cameras with large sensors into TEM has created the opportunity for imaging of larger areas and analysis of serial sections, the acquisition of large-volume morphological information using transmission electron microscopy is extremely labor-intensive and frequently unsuccessful because of section deformation and damage. This limitation can be overcome by using a modern field-emission scanning electron microscope (FE-SEM) with high-sensitivity detection, which enables the creation of TEM-like images from the surface of resin-embedded biological specimens. Several FE-SEM-based techniques for two-dimensional (2D) and three-dimensional (3D) ultrastructural studies of cells, tissues, organs, and organisms have been developed during the 21st century [17,18,19,20,21,22,23,24,25,26,27,28,29,30,31,32]. These techniques have created a new era in structural biology and have changed the role of the scanning electron microscope (SEM) in biological and medical laboratories. Since the premiere of the first commercially available SEM in 1965 [33], these instruments were used in biological laboratories almost exclusively to obtain topographical information over a large range of magnifications. Currently, FE-SEM provides many attractive possibilities in the studies of cell and tissue ultrastructure.

This article provides an overview of FE-SEM-based techniques developed for the ultrastructural studies of large areas (2D) and large volumes (3D) of resin-embedded animal specimens.

## 2. Basic Principles of Image Formation in Scanning Electron Microscopy

**Figure 1:**
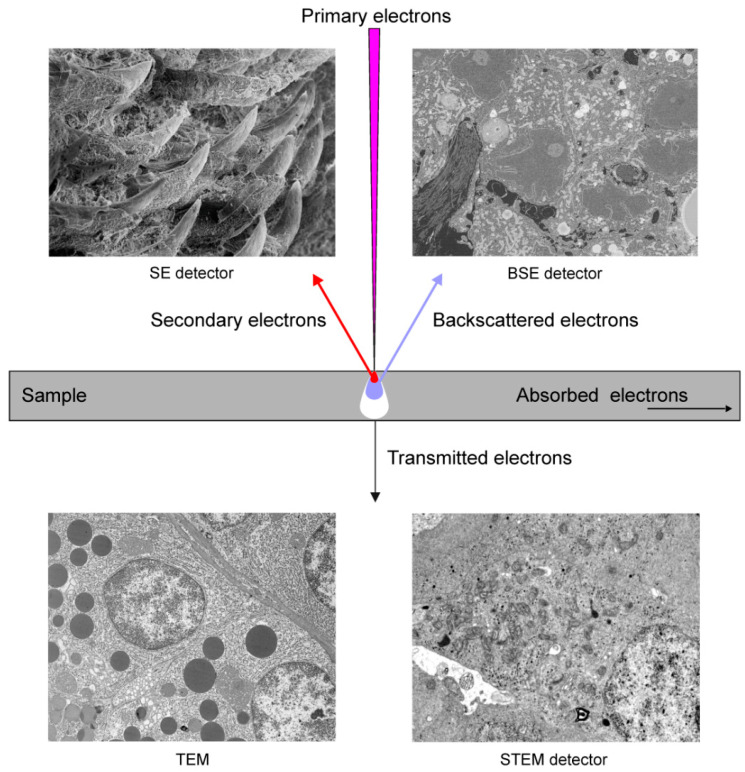
Basic principles of imaging in electron microscopy.

The SEM scans a focused electron beam over the surface of a sample. The electrons in the beam, known as the primary electrons, interact with the sample, producing various signals that can be used to obtain images showing the surface topography and material composition. When the primary electron enters a sample, it frequently travels a certain distance before contacting another particle. After colliding with this particle, the primary electron moves on a new trajectory, which is known as scattering. The entry of the electron beam into the specimen and the scattering events result in the formation of a teardrop-shaped reaction vessel (Figure 1).

Secondary electrons (SE) are generated when the primary electrons extricate the specimen electrons. They have low energy and cannot escape from the deeper parts of the reaction vessel. Therefore, the SE detected in SEM originate exclusively from the surface or the near-surface area of the specimen (Figure 1). The SE produced in the deeper parts of the reaction vessel are absorbed by the sample. The shallow depth of origin of the detected SE makes them ideal for providing high-resolution topographical information. SE production increases slightly with the atomic number of elements; therefore, an SE signal can also be used to obtain TEM-like images from very smooth and flat surfaces of resin-embedded biological samples fixed using heavy metals. The advantage of SE imaging is that the primary electron produces several SE through multiple scattering events, which significantly increases the signal. SE can be detected using Everhart–Thornley, variable pressure SE, and in-column SE detectors.

Backscattered electrons (BSE) are the original beam electrons that escape from the specimen owing to scattering (Figure 1). They have higher energies than SE. BSE emission is a function of the atomic number; therefore, the specimen area containing elements with higher atomic numbers produces a brighter signal. Consequently, BSE can be used to generate an image that indicates the differences in the chemical composition of the sample. The sample volume, from which BSE are generated, is significantly larger than the region that is a source of SE; therefore, BSE have poorer spatial resolution than SE. BSE can be detected using extra column diode-based and in-column detectors. Note that BSE participate to the same degree in the signals delivered by the SE detectors. Moreover, BSE induce the formation of SE.

Since BSE provide information about material composition, they are used as a signal source for imaging of the cut surface of tissue blocks or the tissue sections mounted to the solid substrates in the majority of methods developed to study subcellular and cellular structures of animal tissue, organs, and even entire organisms in 2D and 3D modes [17,18,19,20,21,22,23,24,25,26,27,28,29,30,31,32]. The effective, high-resolution ultrastructural imaging of biological samples using BSE signals is dependent on three aspects. First, the sample preparation procedure should result in the incorporation of a large quantity of heavy metals into the tissue to differentiate cell structures and increase the signal-to-noise ratio. Second, the electron optics of FE-SEM should enable stable imaging at very low acceleration voltages because the size of the reaction vessel is dependent on the acceleration voltage. At higher voltages, the electron beam penetrates deeper into the sample, BSE are emitted from a large volume, and the resolution is lower. Third, highly efficient BSE detectors are required to obtain good signal-to-noise ratios and reasonable acquisition times.

Transmitted electrons are the primary electrons that pass through specimens if they are sufficiently thin (60–200 nm). The transmission of electrons through a sample depends on the atomic number; therefore, osmium fixation and heavy-metal-staining largely increase the contrast of the image. These electrons form images in TEM and can be detected by SEM using a scanning transmission electron microscopy (STEM) detector located under the ultrathin section (Figure 1).

## 3. Multiscale Imaging of Large Sample Areas

The preparation of a resin-embedded tissue block for imaging in TEM frequently involves the cutting of semithin sections (0.5–1.5 μm), which are used to examine a large area of tissue with an optical microscope to locate the regions of interest for ultrastructural studies. Based on these observations, the block is trimmed to a size that enables the preparation of ultrathin sections (50–90 nm), which are placed on mesh grids and then contrasted with heavy-metal salts. Consequently, the investigated area has largely reduced overall dimensions and is divided into separate parts by grid bars, which additionally cover some portion of the sample area. Due to this separation, it is frequently difficult to recognize the histological context of TEM images. These problems can be overcome by the use of single-slot grids with supporting films, which enable obtaining an unobstructed image of the entire ultrathin section. The imaging of the entire ultrathin section or its large part at high resolution in standard TEM is frequently problematic because of the size of sensors in digital cameras and the requirement of a montage of many images into a larger one. A transmission electron microscope camera array (TEMCA) was constructed to increase the image acquisition efficiency in TEM [34], and this idea was recently developed as high-speed TEM [35].

**Figure 2:**
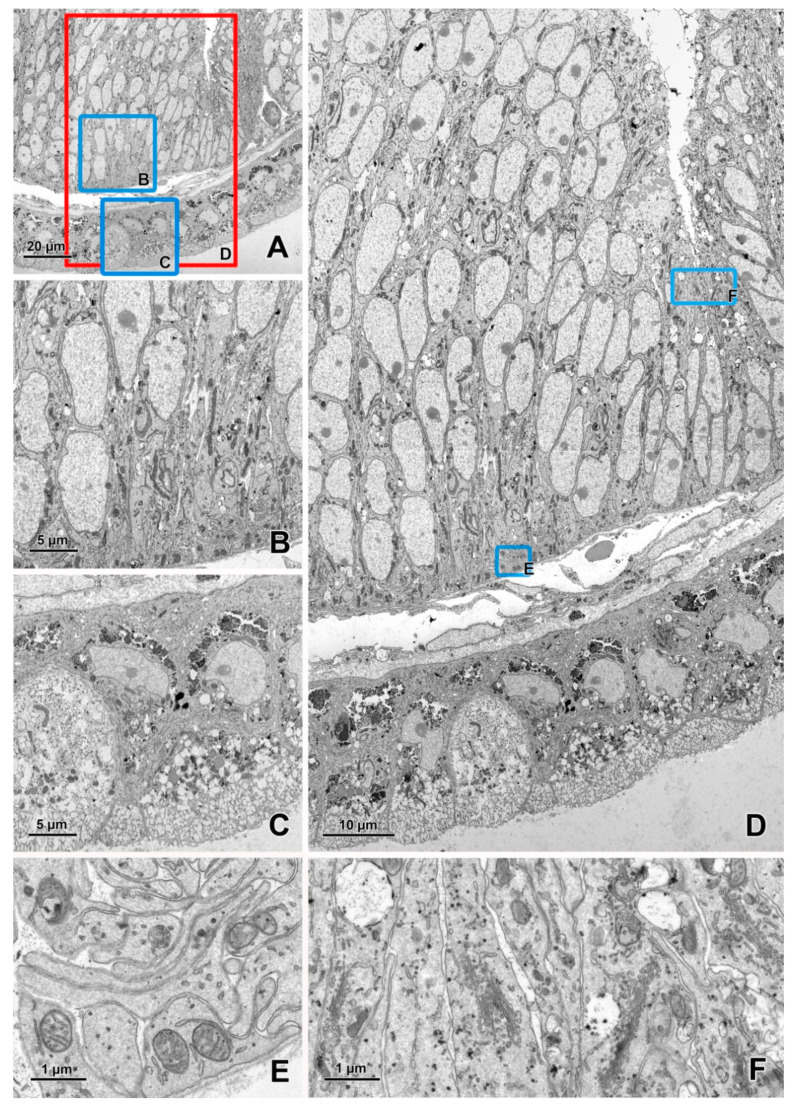
Imaging of an ultrathin section of the river lamprey larvae using a STEM detector. (**A**) An image acquired at a 20 nm pixel size (area 120 × 107 μm). (**B**,**C**) The areas marked by blue rectangles on figure A after zooming in. (**D**) An image of the area (119 × 68 μm) marked with a red rectangle on figure A acquired at a 3 nm pixel size. (**E**,**F**) The areas marked by blue rectangles on figure D after zooming in. The sample was fixed according to the modified protocol by Deerinck et al. [41] and the ultrathin section on a slot grid was imaged using EF-SEM Gemini 450 (Carl Zeiss, Oberkochen, Germany) at 30 kV and a dwell time of 1 μs.

**Figure 3:**
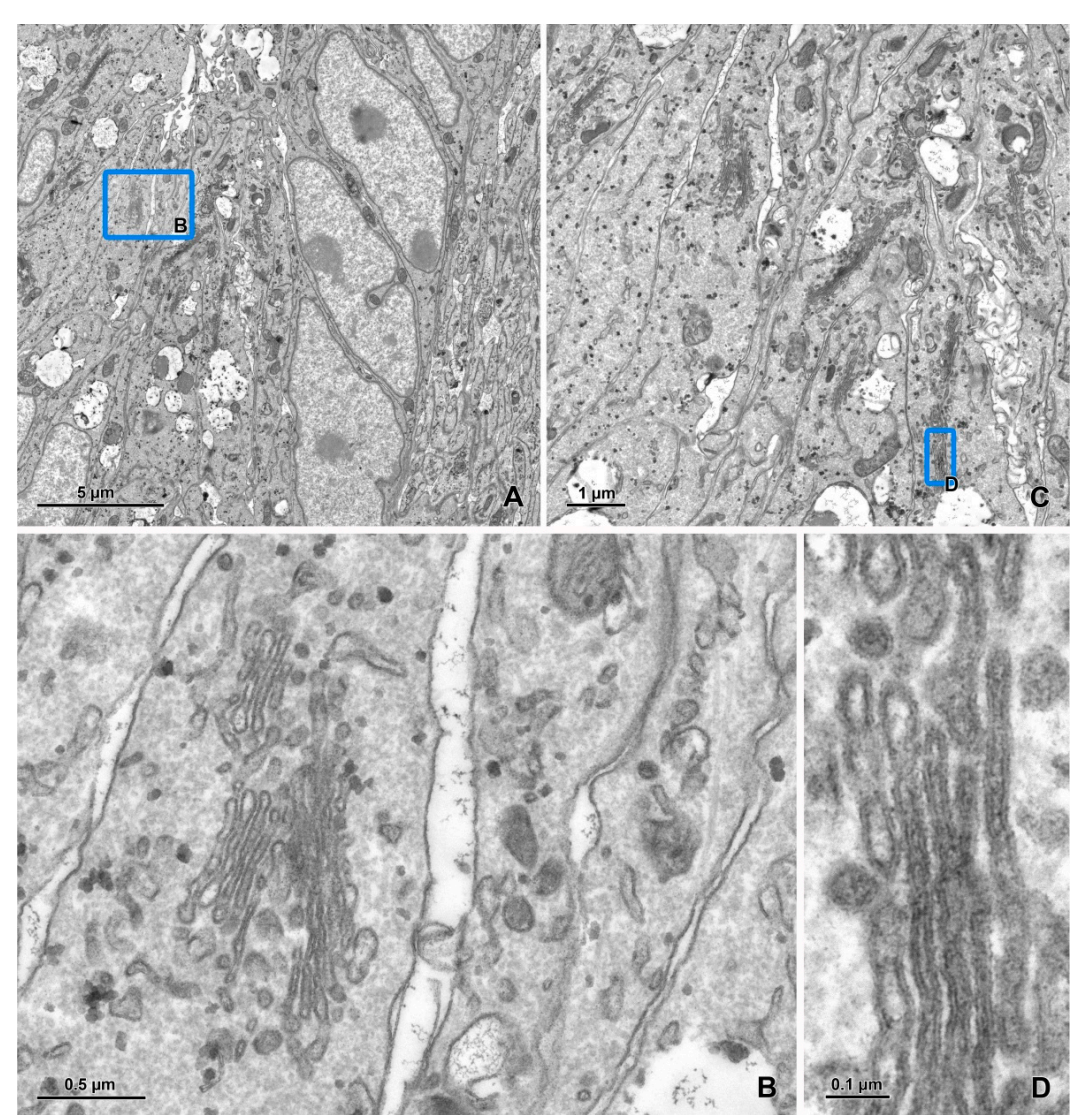
Imaging of an ultrathin section of the river lamprey larvae using a STEM detector (cont.). (**A**) An image of 20 × 21.5 μm area acquired at a 1 nm pixel size. (**B**) The area marked by a blue rectangle on figure A after zooming in. (**C**) An image of 8.7 × 8.5 μm area acquired at a 0.3 nm pixel size. (**D**) The area marked by a blue rectangle on figure C after zooming in. The imaging was performed as described in Figure 2, but with a dwell time of 3 μs.

The detection of transmitted electrons in SEM using a STEM detector is a highly efficient method of imaging ultrathin sections [36,37,38,39]. The electron beam focused on a small spot scans the ultrathin section, and the image is formed by mapping, synchronously with the scan, the signal intensity below the sample. The advantages of this method include automatic imaging of large sample areas with a single frame of up to 24,000–36,000 pixels in each direction (available for many models of SEM), very low noise and high image quality, very high resolution, and significantly shorter acquisition time compared with other SEM methods of TEM-like imaging (Figure 2 and Figure 3). STEM imaging does not require any special staining of samples [38]; furthermore, the contrast in this method is higher than that in TEM because of the lower accelerating voltages (30 kV in SEM vs. 80–120 kV in TEM). Moreover, the transmission mode in SEM exhibits no chromatic aberration. The number of grids loaded simultaneously into the standard SEM holder is 6–12, whereas it is only 1 or 2 in the standard TEM holder. Many commercially available software packages dedicated to SEM provide automatic procedures for STEM imaging, such as overview imaging of all grids, autofocus, and automatic correction of astigmatism. The sections can also be transferred from SEM to TEM if required. Single-slot grids are particularly suitable for STEM detection because they enable an unobstructed digitalization of entire sections [39]. Carbon-coated formvar is frequently used as a support for the sections [39]. Recently, Dittmayer et al. [40] described a workflow to produce high-quality sections on large-slot grids coated with Pioloform films.

The disadvantage of STEM detection as a method of large-area imaging is the necessity of preparing large ultrathin sections on slot grids that require special skills. Another problem is the focal change of the sample signal after longer exposure to the electron beam during image adjustment. This problem could be eliminated by sample pre-irradiation, which can be performed automatically using software macros [39].

**Figure 4:**
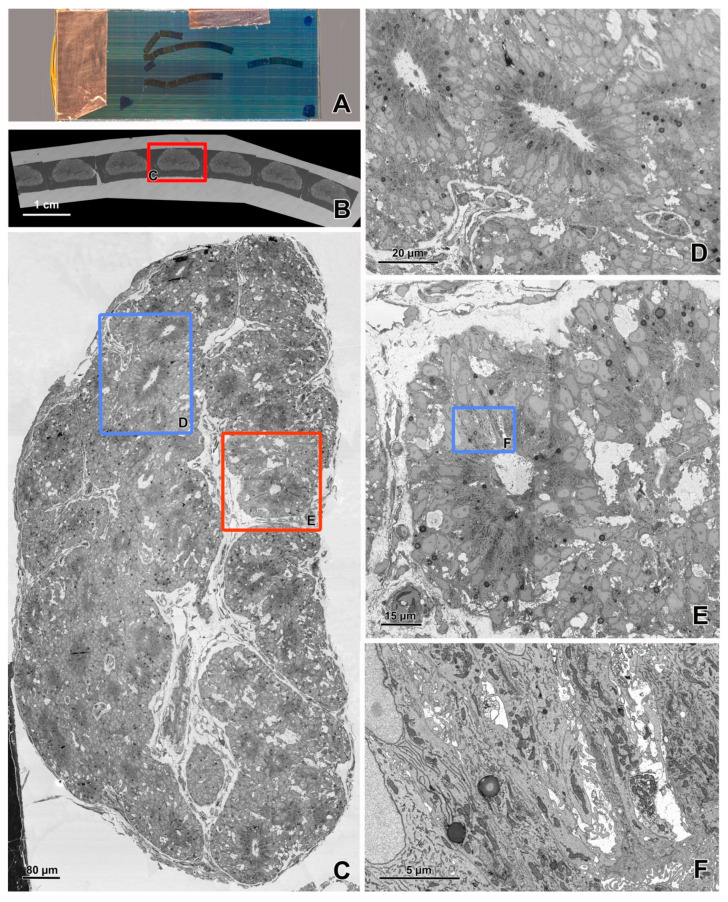
Imaging of ultrathin sections of the embryonic pineal organ of the domestic goose using a BSE detector. (**A**) Macrophotography of section ribbons on the silicon wafer. The arrows show points used for navigation in SEM. (**B**) Overview image of section ribbons acquired using an SE2 detector at a 200 nm pixel size (area 7.4 × 2.2 cm). (**C**) Overview image of a section (area 1080 × 720 μm) marked with a red rectangle in figure B acquired by BSE detector at a 15 nm pixel size. (**D**) Image of the area marked with a blue rectangle in figure C after zooming in. (**E**) Image of the area marked with a red rectangle in figure C acquired at a 3 nm pixel size (area 131 × 132 μm). (**F**) Image of the area marked with a blue rectangle in figure E after zooming in. The sample was fixed according to the modified protocol by Deerinck et al. [41] and the ultrathin sections were imaged using EF-SEM Gemini 450 (Carl Zeiss, Oberkochen, Germany) at 1.5 kV and a dwell time of 1 μs (**B**) or 8 μs (**C**–**F**).

**Figure 5:**
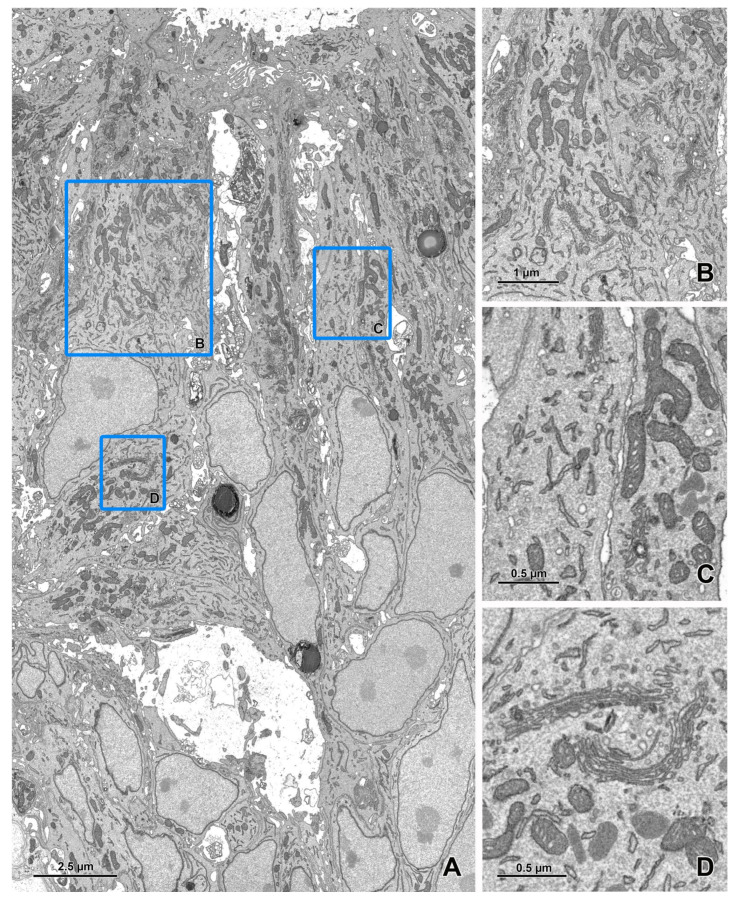
Imaging of ultrathin sections of the embryonic pineal organ of the domestic goose using a BSE detector (cont.). (**A**) An image of a 22 × 13 μm area acquired at a 1 nm pixel size. (**B**–**D**) The areas marked by blue rectangles on figure A after zooming in. The imaging was performed as described in Figure 4 using the BSE detector (dwell time 8 μs).

**Figure 6:**
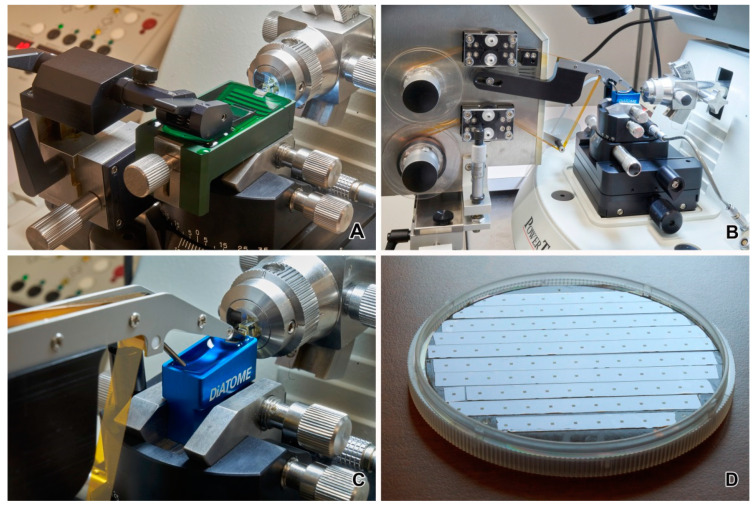
(**A**) Substrate holder for manual collection of sections. (**B**,**C**) Automatic tape ultramicrotome. (**D**) Strips of Kapton tape with sections on 4′ silicon wafer.

BSE detection enables the ultrastructural imaging of sections [17,18,19] placed on solid, electron-beam-impermeable, conductive supporting media, such as silicon wafers (Figure 4 and Figure 5, Supplementary Movie S1). In contrast to the collection of separate sections or very short section ribbons on grids for TEM, the ultrathin sections for imaging using BSE detection are frequently cut into long ribbons using a diamond knife with a large boat (jumbo knife), and these ribbons are attached to a silicon wafer (Figure 4A). A special manipulator is used to hold and move the wafer in a knife boat (Figure 6A). This technique enables the collection of numerous outsized ultrathin sections on a stable support. In contrast to imaging using the transmitted electrons, the semithin sections can be used for BSE imaging [18,19]. The advantages of semithin sections are a larger section area that provides more data about the histological context, compatibility with light microscopy, and a simple technique of cutting and collecting. The disadvantage is a frequently lower surface quality compared with the ultrathin section, and in some scenarios, higher susceptibility to charging. Reichelt et al. [18] and Rodiges et al. [19] demonstrated perfect results of the digitalization of semithin sections through BSE detection for the multi-scale imaging of animal tissues and for pathological diagnostics.

Efficient, high-quality imaging using BSE requires a more intensive infiltration of tissue with heavy metals than for STEM and TEM imaging. Many protocols for sample fixation and en bloc contrasting for BSE imaging [24,41,42,43,44,45,46,47,48,49,50,51,52,53] have been proposed depending on the tissue properties and sample size (Table 1). The sections of tissues fixed with routine TEM methods and contrasted after cutting can also be imaged using BSE detection; however, they frequently require longer acquisition times; therefore, they are less useful for the digitalization of very large sample areas. However, these sections still facilitate better recognition of the histological context of the investigated structure compared with TEM imaging. According to our experience, intensive en bloc heavy-metal infiltration is a technique of choice for BSE imaging because it enables image acquisition at low dwell times, ensures a high signal-to-noise ratio, and eliminates the risk of section contamination during post-sectioning contrasting. The limitation of this procedure is the poor differentiation of chromatin structure and the different appearance of some cell components, such as secretory granules, compared with conventional TEM images.

Silicon wafers provide perfect support for ultrathin and semithin sections because they are highly conductive. The glow discharge treatment of wafers is recommended to obtain a hydrophilic surface, which facilitates the collection of sections. In applications combining light and electron microscopy, indium-tin-oxide-coated glass coverslips or carbon-coated glass coverslips are used to collect ultrathin sections. Histological glass slides coated with 60–80 nm of carbon [18] or flat epoxy resin sheets coated with gold/palladium [19] can be used as a support for semithin sections.

The ultrastructural imaging of resin-embedded biological samples using BSE detection requires a field emission source of electrons (Schottky emitter) that delivers a stable and relatively high current in the electron beam [54]. The beam current should be sufficiently high to provide acceptable image contrast and low noise at short dwell times, but it should not be excessively high. The increase in the beam current increases the acquisition speed; however, it negatively affects the image resolution [54]. The next requirement for SEM is to ensure very small beam diameters on the sample surface at low acceleration voltages. The diameter of the spot at which the electron beam hits the sample is a primary factor determining the resolution of SEM imaging, and a small volume of the reaction vessel ensured by the low acceleration voltage is a crucial factor for the resolution of BSE imaging (Figure 1). The efficiency of the detector is extremely important for high-quality BSE imaging with reasonable acquisition times. Both in-column detectors and retractable diode detectors have been successfully used to ensure a good image quality. Detection systems dedicated to biological samples are highly recommended. The scan generator should enable imaging with a single frame no smaller than 24,000 pixels in the x- and y-directions to decrease the stage movement and the necessity for image tilt montage. The final but very important component of the image acquisition system is software that allows automatic imaging of large sample areas. Research has demonstrated that the signal-to-noise ratio can be significantly improved by applying a negative bias voltage to the sample (beam deceleration) with a simultaneous increase in the acceleration voltage [54,55,56,57]. The increase in the recorded signal occurs owing to the re-acceleration of BSE in the bias field toward the detector. The deceleration retards the electron beam towards the sample and reduces the penetration of the primary electrons into the samples; therefore, a higher acceleration voltage does not result in an increase in the reaction vessel size [54]. For example, when the acceleration voltage is set to 5 kV and the deceleration voltage is set to −3 kV, the landing energy on the specimen surface is 2 keV.

The imaging of large sample areas using a STEM or BSE detector is frequently performed in a hierarchical mode [58], from overview, low-resolution (200–15 nm/pixel) images of the entire sample through middle resolution (10–5 nm/pixel) images of large groups of cells and high-resolution (3–1 nm/pixel) images of small groups of cells or a single cell, to very high-resolution (>1 nm/pixel) images of cell parts and organelles (Figure 2, Figure 3, Figure 4 and Figure 5). Images with lower resolutions are used to locate targets for imaging with higher resolution. For BSE imaging, the pyramid of images is frequently based on digital macrophotography used for navigation and covers a scale from centimeters (length of section ribbons) to nanometers (Figure 4). It should be emphasized that the top of the pyramid of hierarchical imaging frequently contains images as large as 1 Gpixel and their montages. Moreover, several regions of interest can be digitalized with a very high resolution (multi-top pyramid). Large-area imaging enables the creation of a virtual ultrathin slide, which can be zoomed from the millimeter scale to the nanometer scale.

The effective imaging of flat surfaces of resin-embedded biological samples has resulted in the development of 3D imaging techniques, including serial section imaging (array tomography), serial block-face imaging (SBF-SEM), and focused ion beam SEM (FIB-SEM). The last technique requires dual-beam SEM; therefore, it is not presented in this article.

## 4. Array Tomography

The concept of array tomography was proposed by Micheva and Smith in 2007, mostly as a method of high-resolution, volumetric imaging of large numbers of antigens visualized using immunofluorescence [59]. The name “array tomography” was introduced because of the use of serial ultrathin sections to obtain 3D information. The imaging of ultrathin sections enabled the prominent improvement of resolution in the *z*-axis compared with confocal microscopy. Moreover, the cited authors demonstrated that the sections of acrylic resin-embedded tissue could be repeatedly stained with different antibodies, then contrasted with heavy metals and used for ultrastructural imaging in FE-SEM [59]. At the same time, Kasthuri et al. [60] reported the use of automatically cut and collected serial ultrathin sections using a tape ultra-microtome [61] for ultrastructural imaging in SEM to obtain 3D ultrastructural reconstructions. The automation of section preparation highlighted the requirement for the development of high-sensitivity BSE-based imaging systems and automated image acquisition software [27]. As an alternative to the tape ultramicrotome, which at this time was only a prototype, Horstmann et al. [22] described the use of serial ultrathin sections collected on a silicon wafer for ultrastructural volume imaging. The tape ultramicrotome was commercialized by Boeckeler Instruments, Inc., under an early adopters program in 2015. The last achievement in the development of array tomography as a method of 3D ultrastructural studies was the introduction of multi-beam SEM, which largely increased the image acquisition capability [62,63].

The term array tomography, depending on application, comprises three different techniques: (i) fluorescence microscopy array tomography, which delivers volumetric, high-resolution data on the distribution of molecules and enables the detection of several antigens in the same section [59,64], (ii) electron microscopy array tomography, which enables the capturing of ultrathin sections for 3D ultrastructural studies [65], and (iii) correlative array tomography [66,67,68], which combines fluorescence imaging and electron microscopy imaging to obtain voxel-level associations between structure and chemistry. The new concept is the use of array tomography to locate targets for *z*-axis high-resolution imaging through FIB-SEM [69]. Electron microscopy array tomography should be subdivided into the method using a manual collection of sections (Figure 6A) and the method of using an automated tape-collecting ultramicrotome (ATUM). The first method is frequently limited to hundreds of sections in the form of ribbons attached to a silicon wafer, and the second method enables the collection of thousands of sections on a tape (Figure 6B–D). For the first method, the direct location of the section on the silicon wafer frequently eliminates the charging problem and ensures very high-quality imaging; however, it is limited in size owing to the manual collection of sections. These sections are imaged in conventional FE-SEM within a reasonable period, and the size of the data is not extremely large. Recently, new devices for the automatic collection of serial samples on silicon wafers or magnetic tapes have been described [70,71,72].

The use of ATUM enables the automatic collection of thousands of ultrathin sections on tape while they are simultaneously cut with an ultramicrotome (Figure 6B,C). It operates by moving a plastic tape from a supply reel through a tape snout located in a water-filled diamond knife boat to a take-up reel. The sections are collected from the water on the surface to the tape. Glow-discharged Kapton tape is the most commonly used tape in ATUM. After section collection, the tape is cut into smaller strips, which are mounted using double-sided carbon tape to a silicon wafer with a diameter of 100 mm (Figure 6D). Section contrasting on tapes is possible if required. The Kapton tape is nonconductive; therefore, the wafers with tape strips must be coated with carbon to eliminate charging artifacts. However, this coating may be problematic for imaging using SE detection; therefore, the deposition of carbon on the tape before collecting the sections is advised in this scenario. Recently, conductive carbon nanotube-coated polyethylene terephthalate tape has been proposed to eliminate charging problems and increase the imaging quality [73]. Dedicated software is available for imaging serial sections in a semiautomatic manner. The acquisition of very large sets of sections may require several weeks using a single FE-SEM. The time required for the digitalization of large sets of sections can be dramatically reduced using multibeam SEMs, operating with 61 or more electron beams, and enabling the simultaneous imaging of numerous pixels [62].

The most important advantage of array tomography is its non-destructive nature, which enables the reimaging of sections with different resolutions and regions of interest. It is possible to build archives of sections for further studies. The imaging procedure can begin with the digitalization of every n serial sample in an array to obtain a volumetric overview and select the region of interest. Array tomography facilitates a large versatility in terms of the size of the imaged area and resolution. Very high-quality imaging with a pixel size of 1 nm or below is easily achievable for samples placed directly on a silicon wafer. This method enables the study of archival samples fixed with the conventional protocol for TEM, because of the possibility of section contrast. However, note that the best results are obtained after the intensive infiltration of samples with heavy metals because of the lack of charging and section contrasting artifacts. Array tomography enables correlative light and electron microscopy studies, and their combination with 3D analyses [66,68,74]. The disadvantages of this method are the time-consuming preparation of sections for imaging and the alignment of sections into z-stacks.

## 5. Serial Block-Face Imaging

**Figure 7:**
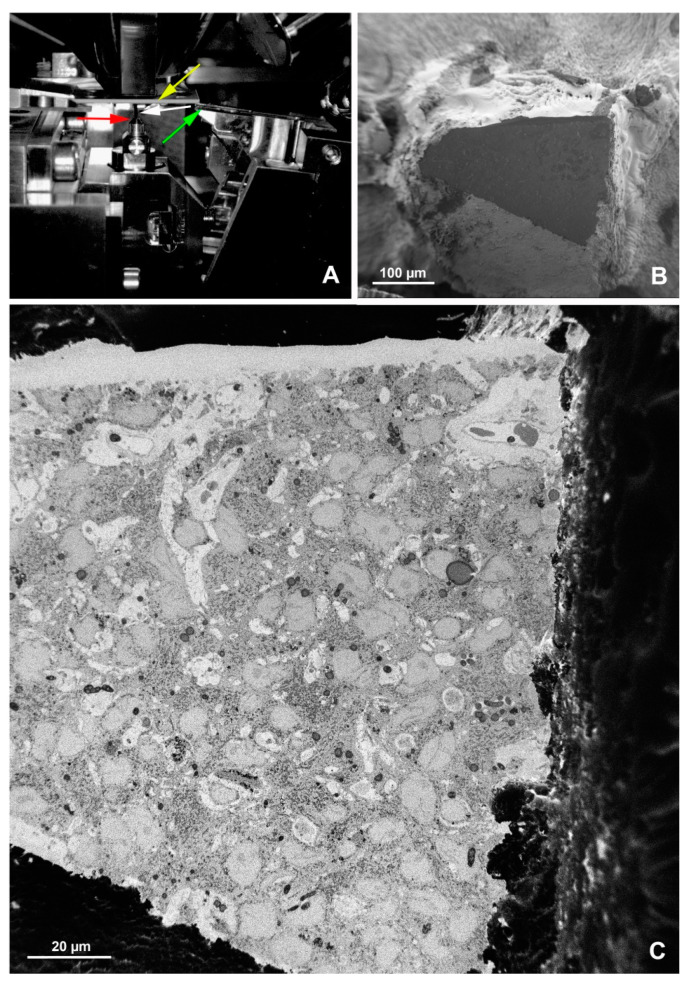
SBF-SEM imaging of the embryonic pineal organ of the domestic goose. (**A**) 3View (Gatan, Pleasanton, CA, USA) in the specimen chamber of EF-SEM Gemini 450 (Carl Zeiss, Oberkochen, Germany). Red arrow—sample, green arrow—knife, yellow arrow—OnPoint detector (Gatan, Pleasanton, CA, USA), white arrow—needle of charge compensation device. (**B**) Block of resin-embedded tissue with visible cutting surface (SE2 detector). (**C**) TEM-like image from the cut surface of the resin block mounted in Gatan 3View. Imaging was performed at 1.2 kV, with a 20 nm pixel size and a dwell time of 2 μs. The sample was prepared according to the modified protocol by Deerinck et al. [41].

**Figure 8:**
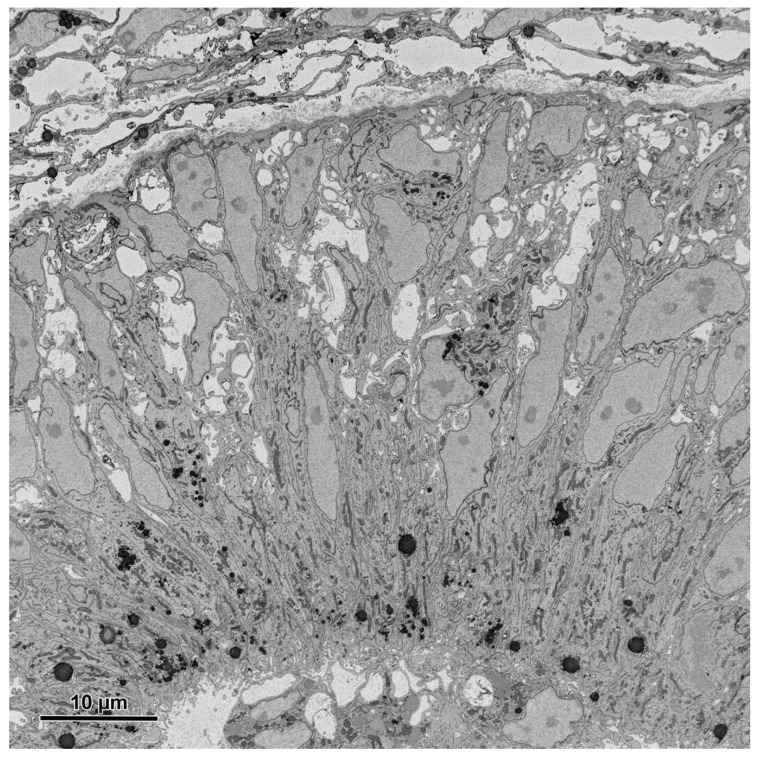
An image (area 64 × 64 μm) of the embryonic pineal organ of the domestic goose obtained using 3View (Gatan, Pleasanton, CA, USA) and OnPoint detector (Gatan, Pleasanton, CA, USA). The sample was prepared according to the modified protocol by Deerinck et al. [41]. Imaging was performed at 1.2 kV, with a 8 nm pixel size and a dwell time of 3 μs.

The SBF-SEM technique is based on removing the top layer of a resin-embedded sample using an ultramicrotome with a diamond knife inside the specimen chamber of FE-SEM and then imaging the exposed surface using a BSE detector (Figure 7 and Figure 8). The steps of cutting and imaging the resin block are repeated hundreds or thousands of times to obtain a z-stack for 3D analysis. The first use of a microtome inside the SEM chamber was reported by Leighton in 1981 [75]; however, the system for automatic block cutting and imaging was constructed more than 20 years later by Denk and Horstmann [20]. This achievement was possible because of developments in SEM and computer technologies. The system of Denk and Horstmann was commercialized by Gatan, Inc., and called 3View (Figure 7). The ultramicrotome in Gatan 3View is mounted to a special chamber door, which replaces the standard door in many SEM models when SBF-SEM is used. It also includes a dedicated high-sensitivity BSE detector. In 2015, FEI Company (now Thermo-Fisher Scientific Inc., Waltham, MA, USA) introduced a microtome for montages on the regular SEM stage in a dedicated FEI microscope. A miniature ultramicrotome for montages on the SEM stage was demonstrated at the Microscopy and Microanalysis Congress in 2019 in Portland, USA, by ConnectomX Ltd. (Grove, Wantage, UK).

**Figure 9:**
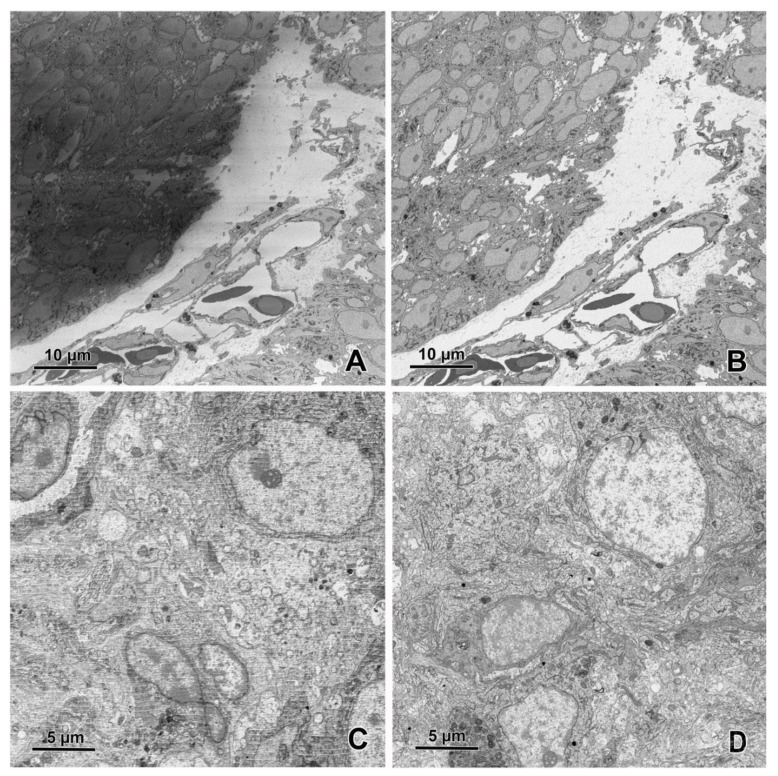
(**A**) Charging on the image obtained using SBF-SEM. Note that artifacts occurred exclusively in one part of the sample. Imaging without focal charge compensation, high vacuum. (**B**) Elimination of charging artifacts by focal charge compensation with 5% nitrogen flow (chamber pressure 4.5 × 10^−4^ mbar). (**C**) Strong damage of surface layer of resin-embedded tissue block caused by the electron beam occurring during cutting at 30 nm thickness. (**D**) The damage is largely reduced after the increase of the cutting thickness to 70 nm. Imaging was performed in EF-SEM Gemini 450 (Carl Zeiss, Oberkochen, Germany) equipped with 3View (Gatan, Pleasanton, CA, USA) and OnPoint detector (Gatan, Pleasanton, CA, USA) at 1.2 kV.

The main advantage of SBF-SEM is the possibility of obtaining a well-aligned z-stack of thousands of images in a fully automatic manner. The setup procedure of SBF-SEM systems is easier and faster than the preparation for imaging in ATUM array tomography, which includes the collection of sections on tape, montage of tape strips on wafers, overview imaging, and selection of regions of interest [65,76]. The SBF-SEM results in a z-stack of images, which may require only a few alignments performed automatically. However, the success of the SBF-SEM method is critically dependent on sample fixation and embedding. The block of the resin-embedded sample must be conductive to avoid charging artifacts and must be resistant to damage by the electron beam (Figure 9). In contrast to section imaging, post-embedding contrasting and carbon coating of the cutting surface are not possible in SBF-SEM; therefore, both the signal-to-noise ratio and conductivity cannot be improved after resin polymerization. Thus, sample preparation must be conducted carefully. The protocol proposed by Deerinck et al. [41] has been successfully used to prepare many types of tissues, but the best results are obtained with the nervous tissue, which is rich in lipids. Adaptations of this protocol to other tissues include various modifications, such as the use of tannic acid and ruthenium red for contrasting collagen or desmosomes [45,77]. Hua et al. [47] proposed a modification that enables the fixation of large samples. Resin embedding and mounting to the pin holder are also important for obtaining satisfactory results [76]. Generally, the resin formulation should ensure the highest hardness after polymerization, and the block must be trimmed to remove the empty resin. For an electrical connection between the sample and stage, the bottom side of the block should contain tissue that is in contact with the sample holder pin, the conductive glue should be used for block fixation to the pin, and the block should be coated with a thin layer of gold.

Three different strategies are used to eliminate the problems caused by tissue block charging during imaging. The oldest one is the use of a low vacuum in the specimen chamber (variable pressure SEM); however, this method induces a large decrease in the signal-to-noise ratio [78]. Despite these disadvantages, this method is commonly used. The second method is conductive embedding, which can be obtained by the addition of carbon particles to the resin or other modifications; however, its effectiveness is currently rather low [79,80]. The third is the focal charge compensation (FCC) proposed in 2017 by Deerinck et al. [81]. FCC is based on the application of nitrogen directly on the block face using a special nozzle during imaging. The dosing of gas is very low; therefore, the high vacuum of the specimen chamber is still maintained (10^−4^–10^−3^ mbar). The locally applied nitrogen gas molecules are ionized, contact the sample surface, and neutralize electrons, which charge the sample. FCC effectively reduces image artifacts (Figure 9A,B); thus, it even enables image acquisition from samples prepared without dense heavy-metal staining. The resolution of imaging with FCC is nearly the same as that without nitrogen application.

The methods based on the mechanical cutting of samples to obtain 3D information have a lower spatial resolution along the z-direction compared with the x- and y-directions because of ultramicrotomy limitations. The microtome effectively removes sections with a thickness larger than 20 nm, whereas the lowest pixel size in the x–y plane is 3–5 nm. As a solution, a deconvolution technique was developed to obtain additional virtual layers in the sample by acquiring images at different primary beam energies and then processing the image stacks using a multi-energy deconvolution algorithm [82,83].

The disadvantages of SBF-SEM are generally opposite to the advantages of array tomography: (i) SBF-SEM is destructive and the tissue cannot be re-examined, (ii) the region of interest is selected based on the first image in the z-stack; therefore, the block of tissue is examined blindly, and (iii) the tissue has to be stained en bloc without an alternative solution. The lateral resolution in SBF-SEM is lower than that in array tomography with sections mounted directly on silicon wafers.

## 6. Conclusions

Modern FE-SEM is a highly powerful and versatile tool for the studies of cell and tissue ultrastructure alone or in combination with the biochemical data provided by fluorescence microscopy. It enables TEM-like imaging of the cut surfaces of tissue blocks, which are impermeable to the electron beam, or ultrathin or semithin tissue sections mounted on the solid substrates, such as a silicon wafer or glass slide. This feature simplifies and accelerates ultrastructural studies of both large areas and volumes of biological samples. However, efficient, high-quality imaging using BSE requires a more intensive infiltration of tissue with heavy metals than for TEM imaging. The imaging of large sample areas in SEM can be performed using two methods based on the detection of BSE or transmitted electrons. The second method is limited to the ultrathin section; however, it provides very high-quality images of conventionally fixed samples. The imaging of large sample areas enables the creation of a virtual slide, which can be zoomed from the millimeter scale to the nanometer scale. Volume imaging techniques comprise AT and SBF-SEM. In AT, serial ultrathin sections are collected manually on a solid substrate or automatically on a tape using a special ultramicrotome. The imaging of serial sections is used to obtain three-dimensional information. SBF-SEM is based on removing the top layer of a resin-embedded sample using an ultramicrotome inside the SEM specimen chamber and then imaging the exposed surface with a BSE detector. This process is repeated to produce a digitized stack of aligned images, which allows to follow cell-to-cell arrangements or intracellular structures in the *z*-direction.

## Figures and Tables

**Table 1:** Protocols of sample preparations.

Authors	Protocol Name	Objective	Primary Fixation	Contrasting En Bloc	Resin	Polymerization	Overall Time ^1^
Seligman et al., 1966 [52]	OTO	- enhancing contrast of lipid- containing membranes and droplets	GA + PFA	2% OsO_4_ in H_2_O	Araldite	nd	nd
				1% TCH (1 h 50 °C)			
				2% OsO_4_ in H_2_O (1 h 60 °C)			
de Bruijn, 1973 [53]	ROTO	- staining of glycogen	3% GA in 0.1 M CB + CaCl_2_ (72 h 0–4 °C)	1% OsO_4_ in 0.1 M CB + 0.05 M K_3_Fe(CN)_6_ (24 h 0–4 °C)	Epon 812	72 h 37 °C, 24 h 60 °C	12 d
Jiménez et al., 2009 [42]	TAMOI	- improving the membrane contrast	2.5% GA, 2% PFA in 0.08 M CB + CaCl_2_ + MgCl_2_ (1 h)	1% OsO_4_ + 1.5% K_4_Fe(CN)_6_ in CB (90 min on ice)	Epon	nd	nd
				1% TA in 0.1 M CB (30 min RT)			
				1% OsO_4_ in H_2_O (30 min on ice			
Deerink et al., 2010 [41]	NCMIR	- enhancing signal for BSE imaging of epoxy-embedded mammalian tissue at low accelerating voltages	2.5% GA, 2% PFA in 0.15 M CB + CaCl_2_ (2–3 h)	4% OsO_4_ + 3% K_4_Fe(CN)_6_ in 0.3 M CB + CaCl_2_ (1 h on ice)	Durcupan	48 h 60 °C	3 d
				TCH (20 min RT)			
				2% OsO_4_ in H_2_O (30 min RT)			
				1% UA in H_2_O (overnight 4 °C)			
				PbAsp (30 min 60 °C)			
Bushby et al., 2011 [43]	-	- enhancing contrast of cells and matrix for visualization through BSE imaging	2.5% GA, 2% PFA in 0.1 M CB (2.5 h RT)	1% OsO_4_ + 1.5% K_4_Fe(CN)_6_ in 0.1 M CB (1 h RT)	Durcupan	24 h 45 °C	2.5 d
				1% TA in H_2_0 (1 h RT)			
Tapia et al., 2012 [44]	-	- high-contrast en bloc staining of neuronal tissue for FESEM	2% GA, 2.5% PFA in 0.1 M CB (1 h RT)	2% OsO_4_ in 0.1 M CB (2 h RT)	Embed 812	48 h 60 °C	4.5 d
				1% TCH (30 min RT)			
				4% OsO_4_ + K_4_Fe(CN)_6_ in 0.2 M CB (1 h RT)			
				LC + CS (2 h 37 °C or overnight 25 °C)			
Starborg et al., 2013 [45]	ROUM	- studying collagen fibril organization	2.5% GA in 0.1 M CB (2 h 4 °C)	2% OsO_4_ + 1.5% K_4_Fe(CN)_6_ in 100 mM CB (1 h RT)	Agar100	72 h 60 °C	5 d
				1% TA in 100 mM CB (2 × 2 h 4 °C)			
				2% OsO_4_ in H_2_O (40 min RT)			
				1% UA in H_2_O (16 h 4 °C)			
Hayworth et al., 2015 [46]	-	- smooth thick partitioning and volume stitching for FIB-SEM imaging	2.5% GA, 2% PFA in 0.1 M PB (2 h RT)	1.5% K_4_Fe(CN)_6_ + 1% OsO_4_ (1 h)	Durcupan	24 h 60 °C	1 d 5 h
				1% OsO_4_ (1 h)			
				1% UA in H_2_O (1 h)			
Hua et al., 2015 [47]	-	- large-volume en bloc staining for electron microscopy-based connectomics	2.5% PFA, 1.25% GA in 0.08 CB + CaCl_2_ (12–24 h 4 °C)	2% OsO_4_ in 0.15 M CB (90 min RT)	Spurr	48–72 h 70 °C	5.5 d
				2.5% K_4_Fe(CN)_6_ in 0.15 M CB (90 min RT)			
				TCH (45 min 40 °C)			
				2% OsO_4_ in H_2_O (90 min RT)			
				1% UA (overnight 4 °C, 2 h 50 °C)			
				PbAsp (2 h 50 °C)			
Mikula and Denk, 2015 [48]	BROPA	- reconstruction of neural circuits	2.5% GA in 0.1 M CB + sucrose (48–72 h 2 °C)	OsO_4_ + K_4_Fe(CN)_6_ + CB + CH_3_NO (96 h RT)	Spurr	48 h 60 °C	26 d
				OsO_4_ + CB (72 h RT)			
				Pyr in H_2_O (72 h RT)			
				OsO_4_ in H_2_O (96 h RT)			
Polilov et al., 2021 [49]	-	- simple preparation of heterogeneous biological samples for 3D-EM	I: 1% GA + 1% OsO4 in 0.1 M CB (40 min 4 °C) II: 2% GA in 0.1 M CB (2 h 4 °C)	2% OsO_4_ in 0.1 M CB (12–20 h 4 °C)	Epon 812	48 h 60 °C	5.5 d
				1% K_4_Fe(CN)_6_ in 0.1 M CB (2 h 4 °C)			
				1% UA in H_2_O (8–12 h 4 °C, 2 h 50 °C)			
				PbAsp (2 h 50 °C)			

^1^ The time from the start of fixation to the end of polymerization; OTO—osmium-thiocarbohydrazide-osmium; R-OTO—ferrocyanide-reduced osmium-thiocarbohydrazide-ferrocyanide-reduced osmium; NCMIR—National Centre for Microscopy and Imaging Research; ROUM—reduced osmium and en bloc uranyl acetate method; BROPA—brain-wide reduced-osmium staining with pyrogallol-mediated amplification; TAMOI—tannic acid-mediated osmium impregnation; RT—room temperature; GA—glutaraldehyde; CB—cacodylate buffer; PB—phosphate buffer; PFA—formaldehyde; TA—tannic acid; TCH—thiocarbohydrazide; UA—uranyl acetate; LC—lead citrate; PbAsp—lead aspartate; CS—copper sulfate; Pyr—pyrogallol; nd—no data.

## Data Availability

Not applicable.

## Supplementary Materials

The following are available online at https://www.mdpi.com/article/10.3390/ani11123390/s1, Movie S1: Imaging of sample on silicon wafer using BSE detector
